# A game-based approach to teaching net and wall games in physical education teacher education

**DOI:** 10.3389/fspor.2026.1854504

**Published:** 2026-07-01

**Authors:** Maximilian Poncet-Rief, Daniel Rode

**Affiliations:** 1 Movement and Sport Pedagogy, Department of Human Movement Sciences, Sport and Health, Graz University, Graz, Austria; 2 Sport Pedagogy and Sport Didactic, Department of Sport and Exercise Science, University of Salzburg, Salzburg, Austria

**Keywords:** badminton, game-based approaches, net and wall games, physical education, physical education teacher education, street racket tennis, teaching styles

## Abstract

Game-based approaches (GBAs) are learner-centered pedagogies for teaching games in physical education (PE) that emphasize developing thinking players through game modification and reflective questioning. GBAs are reported to promote learning across motor, cognitive, and affective domains and closely align with educational aims of many contemporary PE curricula. While GBAs are well-established in some PE contexts, traditional directive and commanding approaches still prevail in others. Physical education teacher education (PETE) is a key site for promoting GBAs as a pedagogical alternative. However, best-practice examples showing how to implement and teach GBAs within PETE remain scarce. This article addresses this issue by presenting the case of one PETE course in Austria in which net and wall (N/W) games were taught both through and about a GBAs. We detail a pedagogical framework of GBAs, the setting, learning objectives, session plans specified through Teaching Styles, and discuss lessons learned. The purpose of this paper is to provide a comprehensive illustration of how, in a cultural context where traditional skill-based approaches remain dominant, a GBA can serve both as method and as content within PETE, offering background and actionable guidance for course design and instruction.

## 1 Introduction

Traditionally, teaching sports and games has been focused on developing learners’ technical skills through decontextualized drills and directive, commanding instruction (1, p. 2). Game-based approaches (GBAs) offer alternative and learner-centered approaches that focus on developing thinking players through modified games and reflective questioning (1–5). Over the past decades, GBAs have become well established in certain cultural and educational contexts, and the body of research on their implementation in physical education (PE) continues to grow.

Although findings are partly inconclusive and often difficult to compare, research suggests that GBAs can promote learning across motor, cognitive, and affective domains – including learners’ skill execution within game play, decision-making, response selection and execution, game involvement, motivation, and enjoyment (6–9). The goals and outcomes of GBAs align closely with the broader educational aims of contemporary PE curricula, with GBAs even informing recent PE curriculum development initiatives (10). Despite an ongoing gap between theory and practical application (9), positive responses of teachers and students have been reported (8) and numerous resources provide guidance and examples of how to integrate GBAs into PE lessons (1, 3, 11, 12).

Despite the increasing international recognition of GBAs, research indicates that traditional teacher-centered, skill- and drill-based approaches continue to dominate in many cultural and educational contexts (13, 14). Physical Education Teacher Education (PETE) programs play a crucial role in shaping future teachers’ instructional beliefs and practices and may therefore serve as key sites for promoting GBAs as pedagogical alternatives. However, examples of how to both implement and teach GBAs within PETE remain scarce.

This article addresses this issue by presenting the case of one PETE course in Austria in which net and wall games (N/W games) were taught through and about a GBA. Specifically, we describe how the course design simultaneously aimed to develop preservice teachers’ understanding of N/W games and of pedagogical principles underpinning GBAs.

The course design was developed and implemented as part of a larger research project on GBAs in PETE. The research design included focus group interviews with students after the conclusion of the course, self-reflective journaling by the educator (first author), and debriefings between educator and the second author after each session. Reflexive thematic analysis (15, 16) was used to analyze the data, focusing on learners’ experiences in relation to the course's learning objectives (see below). The detailed empirical results will be published elsewhere. The purpose of this article is to present and discuss the developed pedagogical course design for others to replicate and adopt, providing a detailed, yet comprehensive illustration of how, in a cultural context where traditional skill-based approaches remain dominant, a GBA can serve both as method and as content within PETE.

## 2 Pedagogical framework: a game-based approach

GBA serves as an umbrella term for different pedagogical approaches and models such as Teaching Games for Understanding (TGfU) (17) or Game Sense (18). Despite their distinct differences, these approaches share some basic pedagogical features such as prioritizing game-like activities (that are progressively modified) over decontextualized drills and accompanying these game-like activities with questioning and dialogue to stimulate problem solving and understanding (1–5, 11, 19).^1^ Following these features, the pedagogical framework of our course design does not adhere to one specific model (such as TGfU), but can be considered a hybrid approach (23) that integrates different GBAs and the Spectrum of Teaching Styles (24, 25). Hybridization – combining models or selected components, potentially yielding new models – has been characterized as an “innovative trend” (23, p. 1058) and is advocated to align pedagogy with contextual demands, enable multi-model and more innovative practice, and capitalize on the complementary strengths of different models (26, 27).

**Figure 1:**
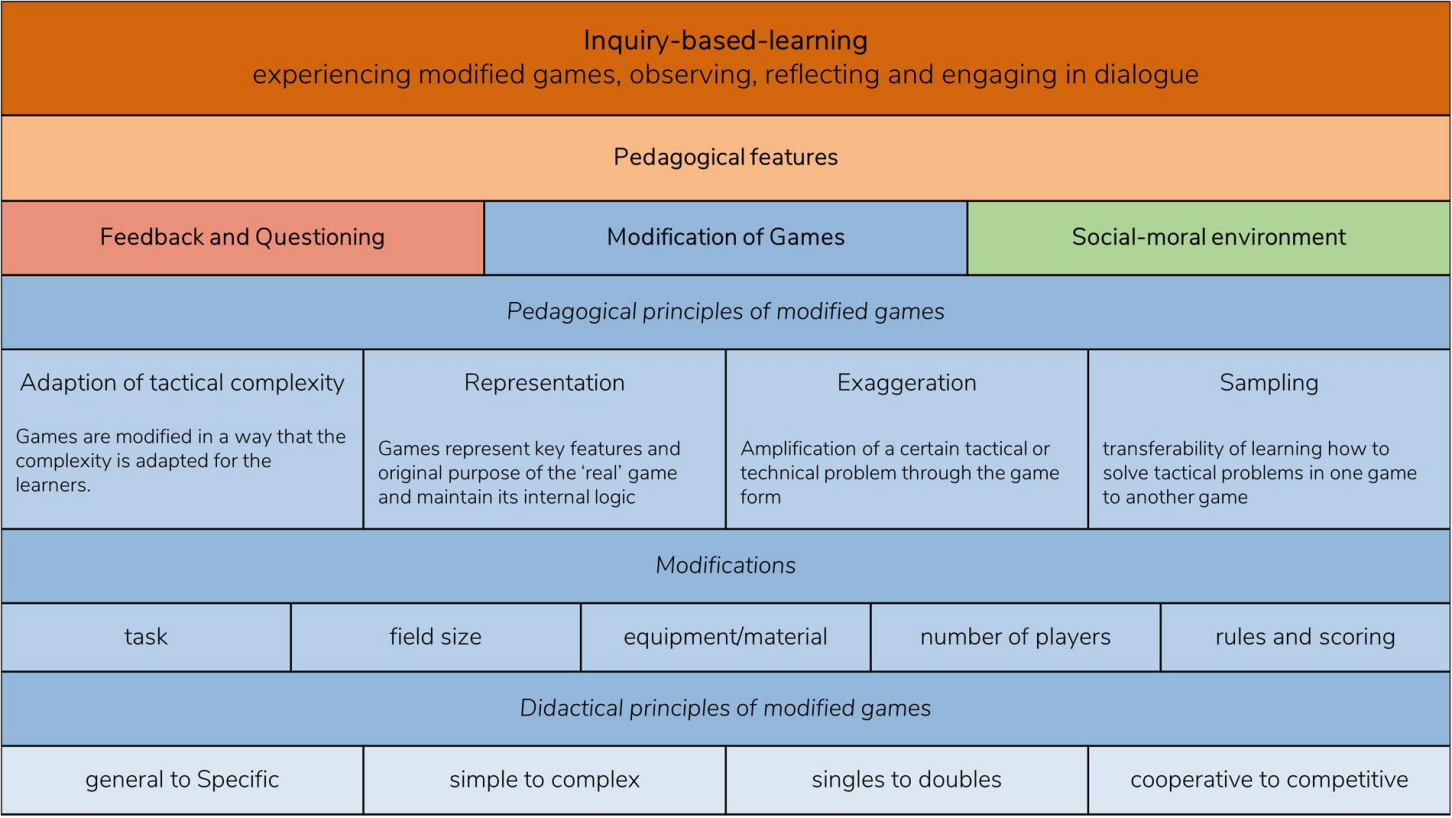
Pedagogical principles and features of modified games.

We tailored our pedagogical framework towards our specific context, target group, and learning objectives (see below). Building on the principle of *inquiry-based learning*, it is shaped by *modification of games*, *feedback and questioning* as well as *anti-hierarchical structures and social-moral environment* as defining pedagogical features (Figure 1) while using the *Spectrum of Teaching Styles* (28) to specify its instructional delivery.^2^

### 2.1 Inquiry-based learning

Inquiry-based learning calls on educators to provide a learning environment which guides students on their own learning journey of exploration (4). Through different modified games, targeted reflections, questioning, and observing, we encouraged students to explore and test different solutions to tactical or technical problems in N/W games. The goal was to enable them to have meaningful learning experiences in and through a GBA, focusing on processes of understanding and taking agency for their own learning process. Thus, combining gameplay activities and reflective discussions with an inquiry-led focus was designed to allow students to learn in and about a game-based pedagogical approach to teaching N/W games.

### 2.2 Modification of games

Traditional approaches often use technical drills as a starting point of learning, advancing to gameplay and tactical concepts only once basic technical skills are acquired in isolation. In contrast to that, our GBA used modified gameplay as the starting point and as dominant form of learning of both tactical concepts and technical skills.

For our gameplay activities, we modified parameters such as field size, equipment, number of players, rules, and scoring opportunities for a N/W game (e.g., badminton) using principles like general to specific, simple to complex, singles to doubles, and cooperative to competitive. The goals were to represent key features of this game while maintaining its internal logic (e.g., striking a ball into the opposing playing field) (feature: *representation*); to allow students to experience gameplay according to their abilities (feature: *adaption of tactical complexity*); to amplify certain tactical or technical problems for the learners to engage with (feature: *exaggeration*); and to show students how learning insights (e.g., using tactical concepts such as depth, width, and positioning to maintain rallies) can be transferred to other N/W games (e.g., racket ball) (feature: *sampling*).

While some GBAs still rely on rather isolated technical drills after introducing technical/tactical problems (18), we rigorously focused on modified game activities. This hinges on understanding technical skills functionally as ‘technique performed in context’ to solve tactical gameplay problems (19), p. 961. For example, asking learners to play badminton on a very narrow and long field led to them engaging with the technical skill of playing a clear or a drop-shot as a possible solution to the tactical problem of creating scoring opportunities through using the concept of depth.

Such activities led to learners wanting to improve a certain technical skill, such as an overhead clear. Sticking to our principle of inquiry-based learning and relying on aspects of a so-called Constraint-Led Approach (CLA) (29, 31–33), we focused on self-exploration and learning through contrasts when addressing such technical learning outcomes.^3^ For example, we asked the students to play clear rallies while using an extremely tight or an extremely loose grip, followed up by targeted questioning and reflection, for them to figure out what kind of grip and use of the wrist works best for using overhead clears to achieve depth in gameplay.^4^

### 2.3 Feedback and questioning

As already implied in the above paragraphs, our approach sides with authors in the field which hold that questioning or a debate of ideas about tactical principles serve as a more fruitful approach to understanding tactical and technical functionalities of games than direct instruction (3, 12, 30). Further, we believe that such questioning increases students’ involvement and engagement in their own learning processes. Asking questions about current learning problems, provoking exploration of gameplay problems, stimulating dialogue among students as well as asking them about how they experienced certain pedagogical principles therefore was a central feature of our pedagogical framework aiming to “promote deep thinking and reflection at an individual and/or collective level” (4), p. 35.^5^

This feature also entailed that feedback was seldom instructional and directive, but instead almost exclusively given in the form of questioning, e.g.,: “What could you have done differently in this situation?”, “What type of shots did you use in this rally?”, “Which other shots might have worked?”. If instructional feedback was given, it was to amplify certain aspects of gameplay or of a particular skill in context and was often linked with questioning, e.g.,: “Try taking your racket further back, so that it almost touches your back. What's the effect of this on your swing?”.

### 2.4 Anti-hierarchical structures and social-moral environment

Tying in with the other features, our pedagogical framework was built towards establishing a positive social-moral environment based on anti-hierarchical structures. It is often stated that GBAs can contribute to positive learning experiences and “affective, personal and social-moral development” (4), p. 14. Achieving this through taking an inquiry-based approach requires an environment that enables and encourages learners to explore, get involved, voice their concerns, make mistakes, and learn from them.

Such a learning environment rests on a culture of constructive mistakes (4), p. 52, where mistakes are considered a natural, integral and constructive part of learning processes. We encouraged learners to test different solutions to gameplay problems, make mistakes, and develop functional solutions based on these experiences.

Encouraging students to start re-shaping their own learning experiences in such a way requires a change of roles of the educator. PE and PETE learning environments often carry hierarchical structures where communication and curriculum are delivered by the educator in a top-down manner. In contrast to that, our pedagogical framework required the educator to take on the role of facilitator of learning: observing rather than interrupting, communicating through questioning, dialogue, and debate rather than through directive instruction. This means “stepping back from center stage” (4), p. 50 to create a collaborative setting between educators and learners that reflects a more equal power relation. We achieved this through rather unformal, very open communication, actively encouraging students to get involved, joint game development as well as actively engaging in game activities when possible or needed.

This change in the role of the educator requires a reflective practitioner who reflects upon his own teaching, continuously questioning his positioning and acting in the group (4). We tried to achieve this through teaching diaries and reflections with a critical friend.

### 2.5 Teaching styles

Many GBAs provide a sound framework and pedagogical principles, but educators lack guidance on how to implement them in their teaching. It has been argued that the Spectrum of Teaching Styles can help provide such guidance (35). The Spectrum of Teaching Styles holds that teaching includes decisions during planning and preparation, during implementation, and during feedback and assessment. Depending on who makes decisions – the educator or the learners – about what and when, different learning objectives can be targeted. The different teaching styles (styles A to K) provide a framework to help educators identify which approach to decision-making responsibilities best corresponds with which kinds of objectives (36, 24).

We relied on The Spectrum of Teaching Styles to specify how our pedagogical framework was delivered in course. To account for the principle of *inquiry-based learning* and the features of *modification of games*, *feedback and questioning*, and *anti-hierarchical structures and social-moral environment*, we mainly relied on teaching styles of the ‘productive cluster’ (styles F to K), which shift decision making to learners to invite productive discovery of new knowledge and skills. Indicated in the session plans below, the specific styles used by us show that different teaching styles can be combined to deliver GBAs (37).

## 3 Setting, students, and faculty

The course “Net and Wall Games” was part of the PETE program at the University of Salzburg and was conducted during the summer term of 2025. It was organized in a block format, comprising a total of 12 contact hours delivered across five sessions ranging from two to three hours in length. Four sessions were held in a large indoor gymnasium, while one session (Session III) took place outdoors on beach volleyball and tennis courts.

The course introduced a variety of N/W games (e.g., wall ball, ring tennis) but focused primarily on badminton – commonly taught in Austrian PE – and street racket tennis, a contemporary N/W game comparable to pickle ball played on tennis or badminton courts with a low net or on improvised surfaces.^6^ It served as a compulsory course within the undergraduate PETE curriculum, designed to develop students’ practical as well as pedagogical competence in the field of N/W games.

Nineteen PETE students participated in the course, representing cohorts from the second to the eighth semester. All students possessed basic knowledge of badminton and demonstrated a corresponding basic skill level (e.g., the ability to maintain a simple rally in badminton). Beyond these shared fundamentals, students’ prior experience with N/W games varied considerably: some were active tennis or fistball players, whereas others had played little beyond school-level experiences.

The course was designed by the first and second authors and taught by the first author, an experienced senior lecturer in the field. In alignment with the pedagogical framework, assessment also followed a GBA. Rather than evaluating isolated technical skills, students’ performance in doubles play in badminton and street racket tennis was assessed according to the criteria of consistency, control, and anticipation.

## 4 Learning objectives

Focusing on learning through and about a GBA to teaching N/W games, the course aimed to develop students’ competence in three interconnected domains: (1) general N/W game competence, (2) sport-specific competence (badminton and street racket), and (3) pedagogical competence related to GBAs.

### 4.1 General N/W games competence

Objectives related to N/W games in general aimed to develop students’ understanding, knowledge, and transferable skills across different games. Specifically, students were expected to:

- know a variety of N/W games and be able to play them in their basic forms
- identify general structures and typical situations of N/W games (e.g., maintaining the object in play, taking the initiative to score, avoiding opponent's score)
- apply core tactical principles, including
  ○ understanding and maintaining effective court position,
  ○ detecting, provoking, and exploiting open spaces to score, and
- using your strengths and opponents’ weaknesses;
- analyze how modifications to key game variables (e.g., playing field, equipment, or rules) influence gameplay dynamics and tactical decision-making across different N/W games.

### 4.2 Sport-specific competence (badminton and street racket)

Objectives related to sport-specific competence addressed students’ understanding and execution of tactical concepts and technical skills within gameplay in badminton and street racket. Students were expected to:

- understand the basic shots in badminton (e.g., clear, drop, smash) and their tactical functions
- develop a functional, individualized technique for these basic shots appropriate to their own skill level
- select and execute appropriate shots during gameplay
- understand tactical concepts such as depth, width, and positioning to maintain rallies, create scoring opportunities, and defend effectively, and apply these concepts in gameplay
- recognize different formations and tactical options in doubles play and evaluate their respective strengths and weaknesses in game situations

### 4.3 Pedagogical competence related to GBAs

Objectives related to pedagogical competence focused on students’ understanding and application of GBAs in teaching N/W games, with an emphasis on reflecting on their own learning experiences. Students were expected to:

- understand the overall pedagogical approach as well as key features and principles of GBAs (e.g., representation, exaggeration, and adaptation through modification of playing field, equipment, task or rules);
- understand the roles of educators and learners in GBA contexts;
- analyze how pedagogical features and educator/learner roles were implemented during the course and reflect on how this shaped their own learning;
- observe and analyze other students’ gameplay using game performance assessment tools, applying these observations to reflect critically on both gameplay and instructional practice.

### 4.4 Learning objectives and the Spectrum of Teaching Styles

To address learning outcomes across the three domains described above, we relied on different styles from the Spectrum of Teaching Styles.^7^ Styles from the reproductive cluster were mainly used either to introduce different games and the session's learning challenges (E-Inclusion) or to strengthen and practice already acquired skills, knowledge, and understanding (B-Practice), as well as to facilitate self- or peer feedback on, for example, game performance or shot selection (C-Reciprocal; D-Self-check). During the main phase of the sessions, discovery styles were emphasized, most commonly “F-Guided Discovery”, especially when game modification and questioning were educator-led and used iteratively to help players explore and make sense of functional shot technique. Additionally, we used “F-Guided Discovery” to address pedagogical competency objectives, such as understanding how game modifications influence play and can be aligned with specific learning outcomes. We then shifted to more open discovery formats in which players had greater latitude to explore options and shape the questioning – for example, investigating how to move the opponent and create space through varied shot selections (G-Convergent Discovery). Where multiple solutions were appropriate, we emphasized exploration and creativity – for instance, when players examined doubles tactics suited to their team or designed their own games and rules using previously learned game-design modifications (H-Divergent Discovery).

## 5 Session plans

This section presents detailed plans for each session, outlining the sequence of learning activities/games, the associated student tasks, and the corresponding learning objectives. For each session, we specify intended learning outcomes at both the session and the activity levels. These connect directly to the overarching objectives within the three domains of general N/W-game, sport-specific, and pedagogical competence described in section 4.

### 5.1 Session I: Basic game-related structures – GBA principles

Session I was a two-hour class that introduced students to the general category of N/W games through wall ball and ring tennis, provided an initial focus on badminton, and familiarized them with the principles of GBAs (Table 1). The intended learning outcomes expected students to:

- understand the general structure and basic tactical principles of N/W games (general N/W games competence)
- identify similarities and differences between N/W games (general N/W games competence)
- develop an initial understanding of basic shots in badminton and their tactical functions (sport specific competence)
- describe basic pedagogical principles of GBAs (pedagogical competence)

**Table 1:** Session plan I basic game-related structures – GBA principles.

Activity	Task and rules	Objective	Teaching style
Wall Ball	Play the ball with one hand so it hits the wall and then the field. Score when the opponent fails to return it. Modifications: number of floor contacts allowed for the ball (rules); using tennis, moon, or other balls (materials); adjusting length and/or width or the field (field); using two walls in a corner (field); variating number of players.	Experience basic tactical structure of N/W games. Explore how modifications affect gameplay.	Inclusion Style-E / Guided Discovery Style F
Ring Tennis (tennikoit)	Throw the ring over the net into the opponent's field. No downplay. Score when the opponent fails to catch it before it hits the ground. You are allowed one step after catching the ring. Modifications: one-handed catches (rules); no entry-zones close to the net (rules/field); singles or doubles play (number of players/rules).	Experience basic tactical structure of N/W Games. Explore how modifications affect gameplay.	
Reflection	What are similarities and differences between wall ball and ring tennis? What are objectives of N/W Games? What is their basic structure? What roles do players take in the game? How can I score? How can I prevent scoring?	Understand structure and basic tactical principles of N/W games based on own playing experiences.	
Badminton	Play badminton with a partner on one half of the badminton court. Start out playing with each other (trying to achieve long rallies) and advance to playing against each other (trying to score). Modifications: narrow and long vs. short and wide field, enlarging or minimizing the field (field size); double points for scoring in the back or front court (scoring).	Explore different solutions for a given tactical problem (using width or length to create scoring opportunities). Experience basic badminton shots (clear, drive, drop).	Convergent Discovery Style-G / Guided Discovery Style-F
Reflection	How were you able to score points on a narrow and long court or on a short and wide court? What kind of shots did you use? Can you name basic badminton shots? How can they be characterized?	Understand tactical concepts of “depth” and “width”. Understand basic badminton shots (clear, drive, drop) and their tactical function regarding “depth” and “width”.	
Pedagogical reflection	Reflective questioning followed by explanation of the pedagogical principles and features applied in this session: What was the basic structure of this session? Which pedagogical principles or features were applied? How did you experience the focus on small-sited games? Which modifications were used – for which purpose?	Describe basic principles of GBAs based on own experiences as learners.	Guided Discovery Style-F

### 5.2 Session II: Understanding basic badminton shots - modification of games

Session II was a two-hour class focused on understanding the differences between basic badminton shots concerning their technical execution as well as their tactical use (Table 2). The intended learning outcomes expected students to:

- know and execute basic shots in badminton and wall ball (sport specific competence)
- identify similarities and differences of different shots in badminton (sport specific competence)
- understand and describe the pedagogical principles adaption, representation, and modification of games (pedagogical competence)

**Table 2:** Session plan II understanding basic badminton shots - modification of games.

Activity	Task and rules	Objective	Teaching style
Wall Ball – Racket Wall Ball	Play Wall Ball according to rules established in Session 1. Modifications: field restrictions on the wall (field size); singles to doubles to large groups (number of players); use of street rackets (material); start every shot from a central position (task). Questioning: What is a functional field position? Where do I stand when attacking and when defending? How can you create a more dynamic game? What's the effect on the game using rackets?	Experience influence of positioning and different shots on gameplay. Explore, develop and understand dynamic gameplay.	Inclusion Style-E / Guided Discovery Style-F
Badminton	Play a clear rally with your partner on a narrow and long court (1/2 badminton court). Modifications: hold racket as tight/loose as possible; hold racket as high/low as possible; hit ball as early/late as possible; make the swinging motion as big and long/small and short as possible (task); If ball comes short (not in the last 3rd of the field) smash (task) Self-Observation: How do different modifications affect your shot? What works best for you?	Experience contrasts concerning technical aspects of badminton shots (grip, swing motion, point of contact). Explore und understand individually functional shot technique.	Guided Discovery Style-F / Self-Check Style-D
Reflection	What do you have to do to shoot the ball for the different badminton shots? Which grip/swing motion/point of contact worked best for you? Why? What are differences and similarities between these shots?	Verbalize individually functional techniques for badminton clears. Understand how technical variations affect the shot.	
Badminton	Clear-Rallye: Who can play the longest overhead clear rally? Modifications: score by smashing the ball as soon as the opponent plays too short (task); variation play: open play trying to score, but you cannot play the exact same shot as your opponent (if ball comes long-longline, play short-longline, short-cross, or long-cross) (task)	Stabilize clear technique. Explore clear variations. Understand how to get opponent moving and create space using variations. Understand interplay of shot variations and tactical concepts (width, length, dynamic).	Convergent Discovery Style-G / Guided Discovery Style-F
Reflection	How did the shot variations help you achieve width and depth? How did they help you manipulate the gameplay dynamic?	Understand interplay of shot variations and tactical concepts (width, length, dynamic).	
Badminton	Open play, try to create scoring opportunities by using shot variations to manipulate depth, width and dynamic of gameplay.	Practice using shot variations to create scoring opportunities. Strengthen understanding of shot variations as solution to tactical problems.	Practice Style-B
Pedagogical reflection	How was today's session organized? What modifications were used? How did they affect gameplay? What were the intended learning outcomes of today's session? What pedagogical principles were used to address these outcomes?	Understand how modifications impact gameplay and can be used to serve specific learning outcomes.	Guided Discovery Style-F

### 5.3 Session III: Similarities and differences of N/W games and beyond – the educator

Session III was a three-hour class that was held outdoors (beach volleyball courts, asphalt area, tennis courts). It introduced large group N/W Games and different forms of street racket to jointly develop a specific form of street racket tennis (similar to pickleball) (Table 3). The intended learning outcomes expected students to:

- develop street racket tennis (sport specific competence)
- experience and understand similarities and differences of different shots in badminton and street racket tennis (general N/W games competence)
- understand similarities and differences in structure and principles as well as the use of modification in N/W games and invasion games (general N/W games competence)
- describe and understand the role of the educator in GBAs (pedagogical competence)

**Table 3:** Session plan III similarities and differences of N/W games and beyond – the educator.

Activity	Task and rules	Objective	Teaching style
*Netball*	Play/throw the ball over the net to the other side of the court; no floor contact; score when opponent fails to return; defend by catching the ball. Modifications: different balls (equipment); play with two or more balls (equipment); one ball to be passed within own team (rules); play volleys only (rules)	Experience large team N/W Games. Experience effect of modifications on large tram N/W games.	Style-E Inclusion
*Street Racket*	Hit the ball into opponent's field; one floor contact; no volley or downplay; score when opponent fails to return. Modifications: vary grip, swing, point of contact (task); two or more bounces (rules); singles to doubles to larger teams (number of players); play on two/four fields (field size/no); play round robin (rules). Self-Observation: explore similarities and differences in shot technique between street racket and badminton.	Experience street racket. Explore similarities and differences between badminton and street racket shots.	Style-G Convergent Discovery / Style-F Guided Discovery
Reflection	How did you strike the ball in street racket? What types of shots did you use? How can they be characterized? What are similarities and differences in shots and shot techniques between street racket and badminton? (e.g., grip, dynamic, racket speed)? How did you score points in street racket? How is this similar or different to netball and badminton?	Differentiate skill and technique within different N/W Games and equipment. Explore similarities and differences concerning shot technique and tactical gameplay.	
Street Racket Tennis	Develop a form of tennis played with street racket materials that fits your group. Fixed rules: play over the tennis net; underhand serve only; score when opponent fails to return. Experiment with modifications to rules and field (e.g., ground contacts, shot restrictions, field size, service box)	Apply modifications for game design. Experience interaction between modifications and structure of N/W games.	Divergent Discovery Style-H / Guided Discovery Style-F
Reflection	Which rules did you agree on for your version of street racket tennis? Which modifications did you try? How did they affect gameplay? How do you think modifications to, e.g., field size affect gameplay in N/W games compared to other games, e.g., invasion games?	Understand interactions between modifications and structure of N/W games. Reflect on similarities and differences between N/W games and invasion games.	
Street Racket Tennis	Play one of your developed street racket tennis variations. You may add variations based on the reflection. Observation task: Use the Game-Performance-Assessment-Instrument (GPAI) to observe gameplay and give feedback to other groups.	Practice street racket tennis play. Observe and give feedback on game performance. Experience and understand understanding GPAI.	Practice Style-B / Reciprocal Style-C
Pedagogical Reflecting	How can we assess good gameplay? What are criteria for good game performance? How were you able to use the GPAI? Based on this and the previous sessions, how would you describe the role of the educator in GBAs? What behavior, skills and knowledge are required?	Understand assessment of game gameplay and the GPAI. Reflect on role of the educator in GBAs.	Guided Discovery Style-F

### 5.4 Session IV: Space, positioning and choosing the right shot – the learner

Session four was a three-hour class introducing fistball and focusing on shot selection and doubles tactics in badminton. It gave students time to practice and further develop skills and knowledge acquired in the last sessions while preparing for the course assessment (Table 4). The intended learning outcomes expected students to:

- explore and understand space and positioning in badminton doubles (sport specific competence)
- learn to choose most functional shots and adapt shot selection efficiently (asport specific competence)
- describe and understand the role of the student in GBAs (pedagogical competence)

**Table 4:** Session plan I basic game-related structures – GBA principles.

Activity	Task and rules	Objective	Teaching style
Fistball	Strike ball with fist or forearm over the net into the opponent’s field; three touches within team with one ground contact in-between; score when opponent fails to return. Modifications: playing on badminton court and volleyball court (field size); play with 2–5 players per team (no. of players); different balls (equipment); after each return come together and clap each teammate hands; two touches per team (rules); play up and down: first set until 11 points, second set winning team plays down from 11 points while losing team plays down from their first set score (rules).	Experience larger team N/W games. Experience effect of modifications in large team N/W games.	Style-E Inclusion
Badminton	1vs1 open play Self-observation: How to alternate shots to score effectively? Observation: Assess others using GPAI or Shuttle-Map and give feedback.	Practice shot selection to create scoring opportunities. Assess gameplay and give feedback	Guided Discovery Style-F / Self-Check Style-D / Reciprocal Style-C
Reflection	Did you use a meaningful variation of shots? How did this help you manipulating width, depth and dynamic to create scoring opportunities? What is important for a good defense? Where and how do I position myself?	Deepening understanding of shot selection as solution to tactical problems.	
Badminton	Doubles open play. Modifications: English double (two pairs playing singles on half court, as soon as one shuttle drops, the game turns into a doubles) (rules); Siamese double (only one racket per team) (rules, equipment); play in a side-by-side, front and back or rotating formation (positioning). Self-observation: Explore advantages and disadvantages of each doubles formation.	Experience different doubles formations. Explore strengths and weaknesses of different doubles formations.	Style-H: Divergent Discovery/ Guided Discovery Style-F
Reflection	How did different doubles formations affect gameplay? What are advantages and differences of these formations? Are there particular game situations where one formation works better than the others? What works best for you?	Understand strengths and weaknesses of different doubles formations. Reflect on which positions and formations work best.	
Badminton and street racket tennis	Doubles open play in badminton and street racket. Experiment with different rotating doubles formations to find a functional solution for your team	Explore doubles formations in two different N/W Games. Consolidate learning understanding of doubles formations, creating scoring opportunities and shot selection	Style-H: Divergent Discovery / Practice Style-B
Pedagogical Reflection	Looking at this and the previous sessions, how would you describe your role as learners? How did you act, what was expected from you?	Understand the role of the learner in GBAs	Guided Discovery Style-F

## 6 Discussion

Research indicates that GBAs can foster motor, cognitive, and affective learning while aligning with the broader educational aims of contemporary PE. However, best-practice examples of how to both implement and teach GBAs within PETE remain scarce. The purpose of this article was to detail the pedagogical framework and course design of one such example developed and implemented for Austrian PETE. Drawing on insights from student focus group interviews, reflective journaling by the first author (the educator) und regular debriefings with the second author – the full results of which will be reported elsewhere – we highlight the following lessons learned and practical implications.

The key lesson is that enabling experience-based learning both *through* and *about* GBAs is the central challenge: addressing and balancing these objectives places high demands on course design, the educator, and the students. As detailed above, we deliberately designed the tasks, sequenced modifications and reflective questioning, and arranged social and organizational structures to target general as well as sport specific N/W games competencies, while simultaneously foregrounding GBA principles. These principles were then made explicit through guided reflection and discussion to support the intended pedagogical learning outcomes.

Students met most of the intended outcomes; group discussions indicated that they appreciated the course, engaged in meaningful learning experiences, and found self-directed exploration of games and game modifications, as well as learning through contrasts and metaphors, especially beneficial.

“At that point, we realized that on a smaller court it’s easier at the beginning. But how does this apply to other sports? We then transferred this idea—for example, in football it’s not necessarily easier on a small field; there you need space and a broader overview. In net/wall games, however, it’s simply easier when you don’t have to cover such large distances. We discussed that quite well, I think, and it makes sense. We also experienced that on a large court it hardly works if you’re not yet proficient, whereas the smaller the playing area, the simpler and more manageable the game becomes.” (Group 1, pos. 38–39)^8^

Reflective journaling and debriefings showed that delivering the course required the educator to adapt session plans responsively, drawing on professionally observing students’ interactions and learning processes, knowledge of guiding pedagogical principles, and deep subject-matter understanding (in this case, across different N/W games).

Thus, the first practical implication is that a deliberate course design and session planning – grounded in a sound pedagogical framework and complemented by the Spectrum of Teaching Styles – provide essential orientation for teaching through and about GBAs in PETE, while requiring professional expertise and adaptive teaching by the educator.

Second, fostering reflection and dialogue warrants particular attention. Reflective journaling and debriefings indicated that the educator found it particularly challenging to pose productive questions that stimulate dialogue and understanding – a well-documented issue in GBA instruction (38). Group discussions suggested that reflecting on their own gameplay was crucial to students’ meaningful learning experiences, yet they also experienced it as challenging and tiring.

“I actually liked it a lot because it’s a different approach […]. But you could tell that during the reflection phases it really depends on everyone contributing at least a little, and that’s sometimes difficult” (Group 1, pos 12)

Consistent with players perspectives in professional sport settings (39), students perceived reflection as less taxing and more meaningful when it was organized as brief, targeted, small-group questioning, paired with game modifications on one or two playing areas. The practical implication is that timing and format of feedback, questioning, and reflection should be calibrated to learners’ needs, with a preference for small-group, on-field reflection and dialogue. At the same time, the potential of more in-depth, whole-group reflection to surface different insights und negotiate shared understanding should not be overlooked.

Third, supporting students in learning *about* GBAs from a pedagogical perspective emerged as the most challenging aspect. Reflective journaling and debriefings indicated that the educator found it difficult to stimulate end-of-session reflection and dialogue in which students discussed their learning experiences, linked them to the GBA principals used, and, as prospective PE teachers, adopted a pedagogical lens. Group discussions suggested that students understood key GBA principles but struggled to transfer them to prospective school PE settings.

“I am super interested in Game-Based-Approaches, but how can we then implement this in different grades? Like, it was good for us, it fit well. But how do I do this in a middle school or maybe in a primary school?” (Group 1, pos. 273)

Prior work suggests that “simply exposing students” to a pedagogical approach is insufficient; content and pedagogical knowledge must be more deliberately intertwined to support understanding and implementation (40), p. 154. A practical response is to integrate brief pedagogical reflections throughout sessions – using microteaching focused on pedagogical competencies (e.g., how the educator organized the learning space) – which in our course was largely placed at the end of class. Barker et al. (41) found that short opportunities for students to evaluate how pedagogical principles might transfer to school settings were beneficial. More broadly, one should not underestimate “the length of time pre-service teachers need to understand various pedagogical models” (42), p. 252. We therefore recommend two ways to embed GBAs more broadly within the curriculum: (1) give students opportunities to experience contrasting frameworks and models within or across parallel courses to facilitate comparison and understanding of their respective strengths (41); and (2) complement practical sessions with accompanying formats that engage students in theoretical discussions of GBAs and the course's pedagogical framework. Positive outcomes have been reported when school-based placements enact game-based pedagogies (42, 43).

These practical implications are drawn from preliminary analysis of our data material. Other constraints of the best-practice example detailed in this article relate to its specific cultural context, target group, course format, and content selection. The course design might require adoption and some of the practical implications might not hold, e.g., when working with students who are more proficient in N/W games; in contexts where GBAs are more (or less) established; in formats with shorter but more frequent weekly sessions; when focusing on other N/W games or different game categories (e.g., invasion games); or when following a GBA that incorporates more isolated skill learning. For such instances, the pedagogical framework, course design, detailed session plans, lessons learned, and recommendations presented here offer concise orientation for teaching through and about GBAs.

## Data Availability

The raw data supporting the conclusions of this article will be made available by the authors, without undue reservation.

## Footnotes

^1^We use the term GBA, as recommended by the Teaching Games for Understanding Special Interest Group (TGfU SIG) (20). For a further elaboration of similarities and differences between GBAs see for example Jarrett and Harvey (21); Renshaw et al. (22).

^2^Our framework mainly draws on the influence of ; González-Víllora et al. (1); Gray (29); Koekoek et al. (3); Light (5); Light and Curry (30); Light and Harvey (4); Renshaw and Chow (31)

^3^The CLA operationalizes ecological dynamics framework and differentiates three different constraints: task, learner, environment. These can be manipulated in different ways, to amplify a certain movement problem.

^4^For differences and similarities CLA and GBA: Renshaw et al. (22). Concerning different forms of feedback in CLA: Otte et al. (34)

^5^For further reading on questioning see: Light and Harvey (4) and González-Víllora et al. (1)

^6^Street Racket materials are available through standard sport suppliers and online shops. However, other N/W games materials (e.g. pickle ball rackets) may also be used.

^7^See Pill et al. (25); SueSee et al. (28) for further elaborations on the different styles.

^8^All direct quotes from the data material were translated from German into English and lightly edited for clarity and readability.

## References

[1] González-Víllora S Fernandez-Rio J Guijarro E Sierra-Díaz JM. Building the game-centred approach. a historical overview, implementatio and transference. In: The Game-Centred Approach to Sport Literacy. Abingdon: Routledge (2020). p. 1–22.

[2] Kinnerk P Harvey S MacDonncha C Lyons M. A review of the game-based approaches to coaching literature in competitive team sport settings. Quest. (2018) 70(4):401–18. 10.1080/00336297.2018.1439390

[3] Koekoek J Dokman I Walinga W. Game-based Pedagogy in Physical Education and Sports: Designing Rich Learning Environments. Abingdon: Routledge (2022).

[4] Light R Harvey S. Positive Pedagogy for Sport Coaching (Second Edition). Abingdon: Routledge (2019).

[5] Light RL. Game Sense: Pedagogy for Performance, Participation and Enjoyment. Abingdon: Routledge (2013). 10.4324/9780203114643

[6] Harvey S Jarrett K. A review of the game-centred approaches to teaching and coaching literature since 2006. Phys Educ Sport Pedagogy. (2013) 19(3):278–300. 10.1080/17408989.2012.754005

[7] Miller A. Games centered approaches in teaching children & adolescents: systematic review of associated student outcomes. J Teach Phys Educ. (2015) 34(1):36–58. 10.1123/jtpe.2013-0155

[8] Oslin J Mitchell S. Game-Centered approaches to teaching physical education. In: Kirk D Macdonald D O’Sullivan M, editors. Handbook of Physical Education. London: SAGE Publications Ltd (2006). p. 627–51. 10.4135/9781848608009.n35

[9] Stolz S Pill S. Teaching games and sport for understanding. Eur Phy Educ Rev. (2014) 20(1):36–71. 10.1177/1356336x13496001

[10] Jarrett K. The utility of game-based approaches within the PE curriculum design and implementation process to develop “more knowledgeable others”. Strategies. (2022) 35(3):3–10. 10.1080/08924562.2022.2052774

[11] Farias C Pill S Griffin L. Game-based Approaches in Physical Education. New York: Routledge. (2025). 10.4324/9781032723327

[12] Griffin L Butler J. Teaching Games for Understanding: Theory, Research and Practice. Champaign: Human Kinetics (2005).

[13] Moy B Renshaw I Davids K. Variations in acculturation and Australian physical education teacher education students’ receptiveness to an alternative pedagogical approach to games teaching. Phys Educ Sport Pedagogy. (2014) 19(4):349–69. 10.1080/17408989.2013.780591

[14] Syrmpas I Digelidis N Watt A. An examination of Greek physical educators’ implementation and perceptions of Spectrum teaching styles. Eur Phy Educ Rev. (2016) 22(2):201–14. 10.1177/1356336X15598789

[15] Braun V Clarke V. Using thematic analysis in psychology. Qual Res Psychol. (2006) 3(2):77–101. 10.1191/1478088706qp063oa

[16] Braun V Clarke V. Thematic Analysis. A Practical Guide. London: Sage (2022).

[17] Bunker D Thorpe R. A model for the teaching of games in the secondary school. Bull Phys Educ. (1982) 10:9–16.

[18] Mitchell SA Oslin JL Griffin LL. Teaching Sport Concepts and Skills: A Tactical Games Approach for Ages 7 to 18 (3rd ed.). Champaign: Human Kinetics (2013).

[19] Barrett L Kinnerk P Kearney PE. Developing skill within the context of a game-based approach. Int J Sports Sci Coach. (2025) 20:960–972. 10.1177/17479541241311673

[20] Teaching Games for Understanding Special Interest Group (TGfU SIG) (2021). Game-Based Consensus Statement. Available online at: http://www.tgfu.info/game-based-consensus-statement.html (Accessed June 05, 2026).

[21] Jarrett K Harvey S. Similar, but not the same: comparing the game based approaches of teaching games for understanding (TGfU) and game sense. Ejournal Rech Interv Educ Phys Sport-EJRIEPS. (2016) 38:92–113. 10.4000/ejrieps.900

[22] Renshaw I Duarte A Button C Chow JY Davids K Moy B. Why the constraints led approach is not teaching games for understanding: a clarification. Phys Educ Sport Pedagogy. (2016) 21:5. 10.1080/17408989.2015.1095870

[23] González-Víllora S Evangelio C Sierra-Díaz J Fernández-Río J. Hybridizing pedagogical models: a systematic review. Eur Phy Educ Rev. (2019) 25(4):1056–74. 10.1177/1356336X18797363

[24] Pill S SueSee B Davies M. The Spectrum of Teaching Styles and models-based practice for physical education. Eur Phy Educ Rev. (2024) 30(1):142–55. 10.1177/1356336X231189146

[25] Pill S SueSee B Rankin J Hewitt M. The Spectrum of sport coaching styles. Routledge (2021). 10.4324/9781003041443

[26] Casey A Kirk D. Models-Based Practice in Physical Education. Routledge Studies in Physical Education and Youth Sport Ser. Taylor & Francis Group (2021).

[27] Fernández-Río J. Another step in models-based practice: hybridizing cooperative learning and teaching for personal and social responsibility. J Phys Educ Recreat Dance. (2014) 85(7):3–5. 10.1080/07303084.2014.937158

[28] SueSee B Hewitt M Pill S. The Spectrum of Teaching Styles in Physical Education. Abingdon: Routledge (2020).

[29] Gray R. How we learn to move: a revolution in the way we coach & practice sports skills. In: Independent (2021).

[30] Light R Curry C. Game Sense for Coaching and Teaching: International Perspectives. Abingdon: Routledge (2021).

[31] Renshaw I Davids K Newcombe D Roberts W. The Constraints-Led Approach Principles for Sports Coaching and Practice Design. Abingdon: Routledge (2019).

[32] Renshaw I Chow J-Y. A constraint-led approach to sport and physical education pedagogy. Phys Educ Sport Pedagogy. (2019) 24(2):103–16. 10.1080/17408989.2018.1552676

[33] Renshaw I Chow JY Davids K Hammond J. A constraints-led perspective to understanding skill acquisition and game play: a basis for integration of motor learning theory and physical education praxis? Phys Educ Sport Pedagogy. (2010) 15(2):117–37. 10.1080/17408980902791586

[34] Otte FW Davids K Millar S-K Klatt S. When and how to provide feedback and instructions to athletes?-how sport psychology and pedagogy insights can improve coaching interventions to enhance self-regulation in training. Front Psychol. (2020) 11:1444. 10.3389/fpsyg.2020.01444 32760314 PMC7371850

[35] Pill S SueSee B. Teaching games and sport for understanding as a Spectrum of teaching styles. In: Pill S Gambles E-AF & Griffin L, editors. Teaching Games and Sport for Understanding. New York: Routledge (2023). p. 108–16.

[36] Mosston M Ahsworth S. The Spectrum of Teaching Styles: From Command to Discovery. New York: Longman (1989).

[37] SueSee B Pill S. Game-based teaching and coaching as a toolkit of teaching styles. Strategies. (2018) 31(5):21–8. 10.1080/08924562.2018.1490233

[38] Harvey S Cushion CJ Massa-Gonzalez AN. Learning a new method: teaching games for understanding in the coaches’ eyes. Phys Educ Sport Pedagogy. (2010) 15(4):361–82. 10.1080/17408980903535818

[39] Kinnerk P Kearney PE Harvey S Lyons M. Gaelic football Coaches’ use of a game-based approach impacts game performance, session characteristics, and player perceptions. Res Q Exerc Sport. (2025) 96(4):732–47. 10.1080/02701367.2025.2496263 40388689

[40] Forrest GJ Wright J Pearson P. How do you do what you do? Examining the development of quality teaching in using GCA in PETE teachers. Phys Educ Sport Pedagogy. (2012) 17(2):145–56. 10.1080/17408989.2011.565470

[41] Barker D Nyberg G Ekberg J-E Larsson H. How does physical education teacher education prepare preservice teachers to make practical judgements when teaching games? A case study. Phys Educ Sport Pedagogy. (2026):1–19. 10.1080/17408989.2026.2662356

[42] Harvey S Cushion C Sammon P. Dilemmas faced by pre-service teachers when learning about and implementing a game-centred approach. Eur Phy Educ Rev. (2015) 21(2):238–56. 10.1177/1356336X14560773

[43] Pill S Penney D Swabey K. Rethinking sport teaching in physical education: a case study of research based innovation in teacher education. Aust J Teach Educ. (2012) 37(8):118–38. 10.14221/ajte.2012v37n8.2

